# Clinicopathological features, response patterns, outcomes and BRAF status in patients with advanced acral melanoma: a preliminary Peruvian study

**DOI:** 10.3332/ecancer.2024.1749

**Published:** 2024-08-27

**Authors:** Denisse Castro, Brady Beltrán, Oscar Carnero, Mauricio Póstigo, Wilhelm Valdivia, Cinthia Figueroa, Manuel Leiva, Marco López, Virgilio E Failoc-Rojas

**Affiliations:** 1 Departamento de Oncología y Radioterapia, Hospital Nacional Edgardo Rebagliati Martins, EsSalud, Jesús María 15072, Lima, Peru.; 2 Centro de Investigación de Medicina de Precisión, Universidad de San Martin de Porres, La Molina 15024, Lima, Peru.; 3 Universidad Católica de Santa María, Arequipa 04013, Peru.; 4 Clinica Valle Sur, Arequipa 04001, Perú.; 5 Departamento de Patología, Hospital Carlos Alberto Seguín Escobedo, EsSalud, Arequipa 04400, Peru.; 6 Departamento de Oncología, Hospital Nacional Adolfo Guevara Velasco, EsSalud, Cusco 80108, Peru.; 7 Departamento de Oncología, Hospital Almanzor Aquinaga Asenjo, EsSalud, Chiclayo 14001, Peru.; 8 Departamento de Oncología, Hospital Nacional Alberto Sabogal Sologuren, EsSalud, Callao 07011, Lima, Peru.; 9 Department of Medicine, Brigham and Women’s Hospital, Harvard Medical School, Boston, MA 02115, USA.; 10 Universidad San Ignacio de Loyola, Lima 15024, Peru; 11 Análisis estadísticos, MedStat-Educación e Investigación, Lima 15072, Peru

**Keywords:** melanoma, cutaneous malignant, treatment outcome, proto-oncogene proteins B-raf

## Abstract

**Background:**

Globally, acral melanoma (AM) is underrepresented in most clinical trials, being predominant in Caucasian populations. Latin America is a niche that needs to be explored. Therefore, this study aimed to determine the clinical features, response patterns, outcomes and v-raf murine sarcoma viral oncogene homolog B1 (BRAF) status in Peruvian patients with advanced AM.

**Methods:**

We retrospectively reviewed the medical records of 19 patients with advanced AM who received immunotherapy (IO) in first- or subsequent-line therapy. The samples were analysed, and their mutational state was performed by deoxyribonucleic acid sequencing, focusing primarily on the most frequently mutated gene, BRAF. Descriptive statistics were used to assess the baseline characteristics. Overall survival was estimated using the Kaplan-Meier method.

**Results:**

The median age was 64 years and 63.2% were men. Plantar was the site most frequently affected (84.2%). The most frequent stage was stage III (68.4%), with 26.4% receiving adjuvant therapy. The majority of cases exhibited a Breslow thickness of >4 mm (52%), a Clark level of IV/V (89.4%), and all patients presented ulceration and a high range of mitosis. During follow-up, all patients experienced recurrent advanced disease, with 52.6% developing visceral metastasis. Patients who received IO as first or subsequent line had an overall response rate (ORR) of 33.3%, and those who received it as first-line therapy had an ORR of 40%. Twenty-one percent of the patients harbored BRAF V6000E mutation and, showing an ORR of 50% compared to wild-type individuals (44.4%) after the first line of treatment.

**Conclusion:**

Our preliminary study reported that AM has poor clinico-pathological features and response rates to IO in Peruvian patients. However, those who received IO as a first-line treatment or harbored the BRAF mutation appeared to have a slightly better response than wild-type patients.

## Introduction

Acral melanoma (AM) is a different subtype of cutaneous melanoma (CM) that originates on the acral surfaces: palms, soles or subungual bed [1, 2]. Its incidence widely varies between different populations. In Caucasians, AM is a rare neoplasia, accounting for 1%–7% of all CM [3]. On the contrary, in countries in Asia and Latin America, AM is the most common subtype of CM. In Asia, it accounts for 47%–86%, while in Mexico it represents 44%, in Colombia 43.7%, and in Peru 35%–61.2% [4–9].

Unlike sun-exposed CM subtypes such as superficial spreading and lentiginous melanoma, AM is rarely related to a precursor nevi or ultraviolet involvement [10]. Furthermore, the Cancer Genome Atlas reported that AM has a much lower mutational burden and frequency of BRAF mutation frequency than other CM subtypes [11–13], which is associated with lower effectiveness of checkpoint inhibitors, such as nivolumab and/or pembrolizumab, compared with sun-exposed CM [14].

Globally, AM is underrepresented in most clinical trials due to the predominant inclusion of study populations of European descent (Caucasian). Furthermore, gene-environment interactions vary between populations, potentially influencing disease development and treatment response [15]. Studies focusing on AM in Latin American populations remain largely unexplored, as research has been conducted primarily in Asian countries. Furthermore, there is a scarcity of published data on AM. Therefore, this study aimed to evaluate the clinical features, response patterns and survival outcomes in a Peruvian cohort of advanced AM. Also, we examined the v-raf murine sarcoma viral oncogene homolog B1 (BRAF) status in our population.

## Methods

### Study population

This retrospective cohort study included all patients with AM diagnoses in five Peruvian national cancer centers from January 2013 to April 2019. The inclusion criteria were as follows: histopathological diagnosis of advanced AM, patients aged ≥18 years, Eastern Cooperative Oncology Group performance status (ECOG PS) 0-2, eligible to receive systemic immunotherapy (IO) for a first or subsequent line of treatment, and clinical history with complete clinical information and follow-up. Patients with a second neoplasm, prior treatment in other healthcare centers, untreated or uncontrolled brain or leptomeningeal metastasis, and insufficient material in skin tissue for next-generation sequencing (NGS) were excluded from the study.

In our cohort, patients could receive IO with nivolumab as the first or subsequent line of treatment (second or third line) according to the protocols established at our institutions in Peru. This means that both treatment-naïve patients and those who had undergone previous chemotherapy were eligible to receive IO with nivolumab.

Approval was obtained from the Institutional Review Board and Ethics Committee of five hospitals in Peru. Data were stored securely and anonymously, and the entire population was included using a census-type sampling approach.

### Variables

The variables included were age, sex, location of the melanoma (palmar or plantar), ECOG PS, clinical stage according to the 8th edition of the American Joint Committee on Cancer, sentinel lymph node biopsy (SLNB), complete lymph node dissection (CLND), involvement of nodular lymphoma, adjuvant therapy, Breslow thickness, Clark level, ulceration, mitosis count, surgical margin, BRAF status, lactate dehydrogenase (LDH) levels, visceral metastases, treatment regimen, overall response rate (ORR), response to IO [complete response (CR); partial response (PR); stable disease (SD); progression of the disease (PD)], progression-free survival (PFS) and overall survival (OS).

### Mutation analysis

Tumour biopsy samples were used for deoxyribonucleic acid (DNA) isolation. A mutational screen was performed, covering a set of 50 frequently mutated genes in solid tumours, using the NEBNext DirectTM cancer hotspot panel obtained from New England Biolabs (Ipswich, MA). After target enrichment, libraries were constructed and indexed according to the manufacturer’s instructions. Subsequently, the libraries were pooled and sequenced at the paired end on Illumina’s miSeq platform (Illumina, San Diego, CA).

Our analysis focuses primarily on the most frequently mutated gene, BRAF. All samples were confirmed for BRAF mutations using Sanger sequencing to ensure optimal sensitivity and specificity in our analysis. The BRAF amplicons were amplified by polymerase chain reaction, purified and subjected to bidirectional sequencing on an ABI3730xl DNA analyzer (Applied Biosystems, Foster City, CA). Chromatogram analysis was performed using Mutation Surveyor Software (Softgenetics, State College, PA).

### Statistical methods

Statistical analyzes were performed with Stata/SE version 16.1 (StataCorp. 2019. Stata statistical software: Release 16. College Station, TX) for Windows 10 Pro x64 bits.

Descriptive statistics were used to assess baseline characteristics, presenting categorical variables in frequencies and percentages, and numerical variables in median and ranges. Kaplan-Meier analyzes were used to determine the OS of patients, and this method was selected to assess the survival function over time. Results are presented in months; all patients were followed for a minimum of 1 year.

## Results

A total of 19 patients aged 18 and over from 5 hospitals in Peru were included: Hospital Nacional Edgardo Rebagliati Martins (Lima), Hospital Nacional Alberto Sabogal Sologuren (Lima), Hospital Carlos Alberto Segun Escobedo (Arequipa), Hospital Nacional Adolfo Guevara Velasco (Cusco) and Hospital Almanzor Aquinaga Asenjo (Chiclayo).

According to the baseline clinical characteristics, 94.7% of the patients exhibited ECOG PS 0-1, the plantar surface being the site most frequently affected (84.2%) followed by the palms (15.8%). All patients underwent primary tumour resection as the definitive treatment; however, approximately 50% underwent an SLNB. Furthermore, the most frequent stage was stage III with 68.4%, and 26.4% of those received adjuvant therapy (Table 1).

According to pathological and molecular characteristics at baseline, most cases exhibited a Breslow thickness of >4 mm and a Clark level of IV/V in 52% and 89.4% of cases, respectively. All patients presented ulceration and a high range of mitosis (≥ 1 mitosis/mm^2^). NGS analysis revealed the presence of the BRAF gene mutation in four cases (21.1%), all of which contained the V600E gene mutation (Table 2).

During follow-up, all patients experienced recurrent advanced disease, with 52.6% developing visceral metastasis. Patients with recurrent advanced AM received first-line treatment as follows: nivolumab 78% (*n* = 10), chemotherapy 21.1% (*n* = 6) and palliative radiotherapy 28.6% (*n* = 3). Overall, patients who received IO as the first or subsequent line had an ORR of 33.3% and those who received it as first-line therapy had an ORR of 40%. Among those who harbored the V600E mutation (*n* = 4), with two patients receiving IO, the ORR was 50%, compared to 44.4% in wild-type patients Table 3.

**Figure 1.**
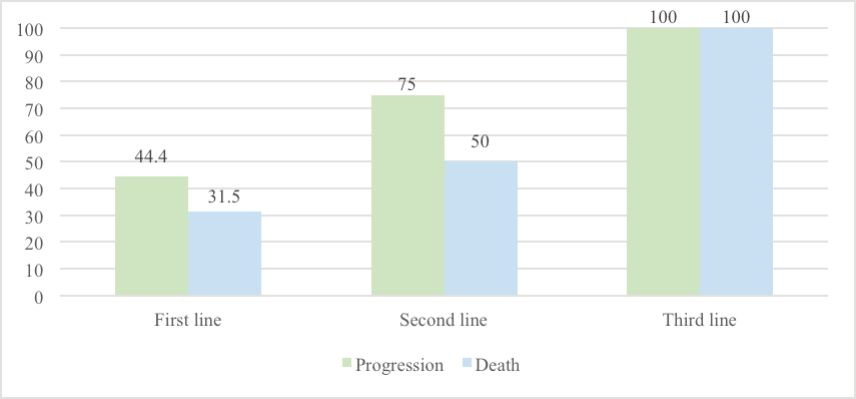
Progression and mortality rate in advanced AM based on the timing of IO introduction.

It was observed that as nivolumab use was introduced later, the progression rate increased (the progression rate in the first, second and third lines was 44.4%, 75% and 100%, respectively). Furthermore, the mortality rate was 31.5% for those who received nivolumab as a first-line treatment compared to those who received it as a subsequent-line treatment (second line: 50% and third line: 100%) Figure 1.

**Figure 2.**
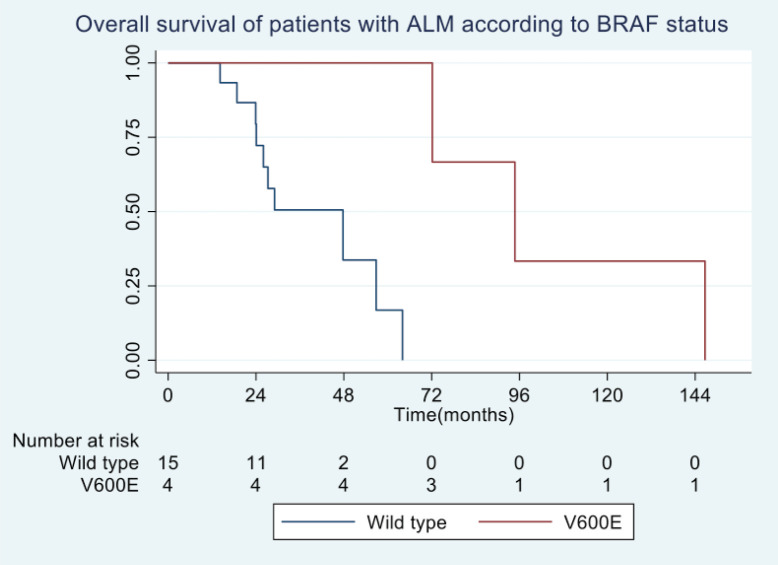
OS in patients with advanced AM according to BRAF status.

In general, the median OS of all patients was 55.5 months. The OS for patients with BRAF V600E mutation was 94.5 months, compared to 29.5 months for wild-type patients (Figure 2). No differences in survival were observed according to sex. The median OS in clinical stage II was longer than in stage III at diagnosis (56 months versus 46 months).

## Discussion

In our study, we found that our population had poor clinico-pathological features, poor response to IO, and a prevalence of 21% of BRAF mutations. However, those who received IO as a first-line treatment or harbored the BRAF mutation appeared to have a slightly better response than wild-type patients.

Overall, our results confirmed that the plantar site was the most frequent location, similar to findings from previous studies conducted in Latin American and Asian populations [5, 16–18]. Additionally, our patients exhibited more advanced disease, and poorer pathological features. This aligns with other studies that found that Asian and Hispanic patients exhibited more advanced disease staging [5, 17, 18], thicker Breslow depth [7, 17, 18] and more ulcerative lesions [5, 7, 17, 18] compared to Caucasians [19].

In addition, we found that patients with advanced AM who received nivolumab in the first or subsequent line had a modest response, with an ORR of 33.3%. However, patients who received it as a first-line treatment achieved a higher ORR of 40%. This is similar to data published in the United States, which reported an ORR of 32% in first-line treatment with monotherapy using nivolumab or pembrolizumab, with a median OS of 31.7 months [20]. However, our results were higher than those of Asian studies from Japan and China, which reported ORRs ranging from 16.5% to 18.8% [21–23]. A larger study from Japan with 193 AM patients found that monotherapy with nivolumab or pembrolizumab achieved an ORR of 16.5% and a median OS of 18.1 months [22]. Nevertheless, a Japanese study showed a slightly improved result of 42.9% with a combination of anti-PD1 and anti-CTLA-4 in patients with AM [24]. This suggests that the efficacy of IO in AM might be influenced by ethnicity.

Moreover, our study revealed that patients treated with nivolumab as a front-line therapy exhibited superior outcomes compared to those treated in subsequent lines. Additionally, delayed initiation of correlated with increased rates of disease progression and mortality. These findings are consistent with existing literature, which shows the 2-year survival probability was higher with first-line therapy (0.5) compared to second-line (0.26), and third-line (0.14) therapy in advanced CM [25]. Thus, initiating IO in the first-line setting is recommended over subsequent lines, for both AM and sun-exposure CM.

Our main findings can be well explained by previous reports that found income, education, and social welfare might contribute to delayed diagnosis in these populations, leading to poorer clinical features and prognosis compared to Caucasian patients [26]. These differences between populations reflect the complex etiology of the disease, which includes the existence of different ethnic groups within these populations and the disparity in healthcare access [27].

Also, we found that the BRAF V600E mutation (21%) was present in the overall population. The frequency of BRAF mutations in AM is low and has been underreported compared to CM exposed to the sun, exhibiting differences between Caucasian, Asian and Latin American countries [28–34]. In Caucasian populations, the frequency of BRAF mutation in AM has been reported to range from 8% to 15% [20, 28, 29]. However, a recent and larger study from the United States determined a higher frequency of BRAF mutations (21.3%), which was similar to our findings. In Asia, the prevalence of the BRAF mutation varied between countries, with a frequency of 13.8%–15% reported in China [31, 35], Korea, 19.4% [32], and India, 31% [33]. On the other hand, Brazil reported a frequency of 31% BRAF V600E/V600K mutations among all AM cases [34]. The contrast in BRAF frequency among populations denotes clear ethnic and racial differences between Caucasian, Asian and Hispanic populations.

Interestingly, although the assessed population was small, patients with AM who harbored BRAF mutations showed slightly better results (ORR: 50%) compared to those with wild-type (ORR:44%), suggesting a potentially greater benefit from IO comparable to sun-exposed CM. In contrast to our results, a Japanese study reported a higher ORR in BRAF wild-type AM patients compared to those with BRAF mutations after anti PD-1 monotherapy (20.7% versus 8.1%; *p* = 0.04) [36]. Similarly, Shoushtari *et al* [20] in the United States found that patients with BRAF mutations did not respond to anti PD-1 monotherapy. However, a more recent study with a larger patient cohort, including AM (11.5%), found that the combination of anti-LAG3 and nivolumab had a better median PFS compared to nivolumab monotherapy (10.6 versus 4.6 months), regardless of BRAF mutation status [37].

Our study had limitations. Specifically, it was a retrospective study with a small sample size of patients with AM. However, our aim is to improve the reliability and generalisability of our findings by incorporating more patients in future prospective cohort studies. The challenge of conducting analyses with small sample sizes is that they increase the risk of Type II errors, reducing statistical power to identify significant differences between groups or to perform subgroup analyses. Additionally, anti-BRAF therapy was not available in our institution for those with BRAF mutations, preventing us from testing its efficacy in our study population.

Additionally, our study provides valuable information and raises awareness about the influence of race/ethnicity, mutational status, timing of treatment delivery and efficacy in AM. More research should be conducted with a larger sample size in a multicenter study in Latin American countries.

In conclusion, our study showed that Peruvian patients with AM have poor clinico-pathological features, and limited response to IO. We found a BRAF mutation prevalence of 21% among our cohort. Also, patients treated IO as first-line therapy or those with BRAF mutations showed slightly better responses compared to wild-type patients. Nevertheless, there remains a critical need for a deeper understanding of this distinct disease. Collaborative efforts involving larger, comprehensive prospective studies across Latin American countries are crucial to fully elucidate the genetic, molecular, and ethnic factors influencing treatment patterns and survival outcomes in AM.

## Conflicts of interest

None of the authors had personal, financial, commercial, or academic competing interests.

## Funding

There was no financial support for this research.

## Availability of data and materials

All data generated or analysed during this study are included in this article.

## Informed consent

This study was carried out in accordance with the ethical standards of the responsible institution for human subjects, as well as with the Declaration of Helsinki.

## Ethics approval and consent to participate

This study was carried out in accordance with the Declaration of Helsinki. The approval was obtained from the Institutional Review Board and Ethics Committee of each institution for the use of skin samples and data. Written informed consent was obtained from all participants. This data was securely and anonymously stored.

## Consent for publication

The manuscript has not been submitted for publication or consideration elsewhere.

## Author contributions

Conceptualisation: DC. Methodology: DC and VEFR. Initial manuscript draft: DC. Data analysis: VEFR. Interpretation: All authors. Writing – Review and editing: DC and VEFR. All authors read and approved the final version of the manuscript.

## Figures and Tables

**Table 1.** Baseline clinical features of AM.

Patient features	*N* = 19 (100%)
Age-median (range)	64.5 (41–82)
Sex	
Female	7 (36.8%)
Male	12 (63.2%)
Localisation	
Palmar	3 (15.8%)
Plantar	16 (84.2%)
ECOG	
0–1	18 (94.7%)
2	1 (5.3%)
SLNB	
Negative	3 (27.3%)
Positive	8 (72.7%)
CLND	
No	5 (26.4%)
Yes	14 (73.6%)
Lymph node compromise	
No	6 (31.6%)
Yes	13 (68.4%)
AJCC stage (8th edition)	
IA	1 (5.3%)
IIA	1 (5.3%)
IIB	3 (15.7%)
IIC	1 (5.3%)
IIIB	1 (5.3%)
IIIC	8 (42.1%)
IIID	4 (21 %)
Adjuvant therapy	
No	14 (73.6%)
Yes	5 (26.4%)

Abbreviations: ECOG: Eastern Cooperative Oncology Group; SLNB: Sentinel lymph node biopsy; CLND: Complete lymph node dissection; AJCC: American Joint Committee on Cancer

**Table 2.** Baseline pathological and molecular features of AM.

Patient features	*N* = 19 (100%)
Breslow thickness	
Median (range)	9.8 mm (40–0.3)
Clark	
I–II	1 (5.3%)
III	1 (5.3%)
IV	9 (47.3%)
V	8 (42.1%)
Mitosis count	
N° mitoses, (range)	3.4 (1–10)
Surgical margin	
Negative	18 (94.7%)
Positive	1 (5.3%)
BRAF status^a^	
Wild type	15 (78.9%)
Mutation^b^	4 (21.1%)

a Abbreviations: BRAF: B-raf proto-oncogene, serine/threonine kinase

b Braf V600E mutations were present in four cases

**Table 3.** Clinical features and treatment outcomes at recurrent advanced AM.

Patient features	*N* = 19 (100%)
LDH (at recurrence)	
Normal	8 (42.1%)
High	8 (42.1%)
Unknown	3 (15.7%)
Visceral metastasis	
No	9 (47.3%)
Yes	10(52.6%)
ORR to IO as first or subsequent line^a^	15(100%)
CR	1 (6.7%)
PR	4 (26.6%)
SD	3 (20%)
PD	7 (46.7%)
ORR to IO as first line^a^	10 (100%)
PR	3 (30%)
CR	1 (10%)
SD	2 (20%)
PD	4 (40%)
Death	
Yes	13 (68.4%)
No	6 (31.6%)

a Response was evaluated according to the response evaluation criteria in solid tumours (RECIST version 1.1)

Abbreviations: IO: Immunotherapy; ORR: Objective response rate; CR: complete response; PR: partial response; SD: stable disease; PD: progression of the disease
